# The legacy of the COVID-19 pandemic on critical care research: A descriptive interview study

**DOI:** 10.1177/17511437241301921

**Published:** 2024-12-08

**Authors:** Natalie A. Pattison, Geraldine O’Gara, Brian H Cuthbertson, Louise Rose

**Affiliations:** 1 School of Health and Social Work, University of Hertfordshire, Hatfield, Hertfordshire, UK; 2 East and North Herts NHS Trust, Stevenage, Hertfordshire, UK; 3 Imperial College London, Imperial College Healthcare NHS Trust, London, UK; 4 Kings College London, London, UK; 5 The Royal Marsden NHS Foundation Trust, London, UK; 6 Department of Critical Care Medicine, Sunnybrook Health Sciences Centre, Toronto, ON, Canada; 7 Department of Anesthesiology and Pain Medicine, University of Toronto. Toronto, ON, Canada; 8 Florence Nightingale Faculty of Nursing, Midwifery and Palliative Care, King’s College London, London, UK

**Keywords:** Research, clinical activity, trials, infrastructure, pandemic, COVID-19, qualitative

## Abstract

**Background:**

The COVID-19 pandemic challenged both research and clinical teams in critical care to collaborate on research solutions to new clinical problems. Although an effective, nationally coordinated response helped facilitate critical care research, reprioritisation of research efforts towards COVID-19 studies had significant consequences for existing and planned research activity in critical care.

**Aims:**

Our aim was to explore the impact of the COVID-19 pandemic research prioritisation policies and practices on critical care research funded prior to the pandemic, the conduct of pandemic research, and implications for ongoing and future critical care research.

**Methods:**

We undertook a descriptive qualitative study recruiting research-active clinician researchers and research delivery team members working in critical care. We conducted digitally recorded, semi-structured interviews in 2021–2022. Framework Analysis was used to analyse the data.

**Results:**

We interviewed 22 participants comprising principal investigators, senior trial coordinators and research delivery nurses from across the UK. Six themes were identified: *Unit, organisational and national factors; Study specific factors; Resources; Individual/clinician factors; Family/patient factors; Contextual factors.* These themes explained how a nationally coordinated response during the pandemic affected individuals, studies and wider organisations in managing the research response in critical care, highlighting future implications for critical care research.

**Conclusion:**

Harnessing the collective response seen in the COVID-19 pandemic in critical care could better support integration of research activity into routine critical care activities. Future endeavours should focus on workforce preparations, contingency planning, strategies for study prioritisation and integration of research as part of the continuum of clinical care.

## Introduction

Rapid global expansion of critical care services during the COVID-19 pandemic became the focus of significant public attention.^1^ The pandemic created an urgent need for research to understand COVID-19 pathophysiology, and to identify effective new treatments to reduce mortality and morbidity. In the UK, and internationally, several clinical trials and cohort studies were established rapidly to study the disease and identify optimal treatments. Examples include studies focused on understanding genetic susceptibilities (e.g. GenoMICC^2^), and treatment-focused platform trials (e.g. REMAP-CAP^3^ and RECOVERY^4^). Many resources were swiftly diverted to support this research activity, with every hospital in the UK strongly encouraged to participate in these studies using the National Institute for Health and Care Research (NIHR) resources.

To guide research activity prioritisation in the UK, an urgent, government-driven response was developed.^5^ Led by the UK Department of Health and Social Care (DHSC), this response included an Urgent Public Health (UPH) portfolio adoption process. The UPH portfolio prioritised COVID-19 studies and ongoing studies that were amended to include COVID-19 treatments, or oriented to understanding more about COVID-19. Once approved, this process enabled prioritised studies to be processed very rapidly through local research sponsorship application, national research ethics committee review, and local recruiting site approval processes. The DHSC strategy also included the release of rapidly convened funding opportunities targeted at COVID-19 research. Similar processes were adopted in other parts of the world, including by the FDA in the US.^6,7^

Reprioritisation of research efforts towards studies deemed important during the COVID-19 pandemic, while essential, had significant consequences for existing and planned research activity in critical care. Many active studies were suspended as were studies with funding approved but not yet activated.^8^ Studies not accepted to the UPH portfolio were deprioritised and thus unable to receive research sponsorship and research ethics committee approval. Furthermore, there was a refocusing of resources not only to UPH prioritised research activity but also to the delivery of clinical care resulting in the redeployment of research staff.^8^ The deleterious effects of COVID-19 on research activity prioritisation away from other disease areas such as cancer treatment,^7,9–12^ and non-COVID-19 critical care research during the pandemic are well documented, but there is little empirical research on the lasting effect on critical care research following the COVID-19 pandemic. It is important to understand the factors that influenced decision-making with regard to prioritisation of research activity, as well as understanding the short and medium-term effects of COVID-19 on the design, conduct and completion of research in critical care and lessons learned. This understanding will inform more effective planning and protection for non-pandemic critical care research and research during future pandemics. For these reasons, we conducted a descriptive qualitative interview study with the aim of exploring the impact of the COVID-19 pandemic research prioritisation policies and practices on critical care research funded prior to the pandemic, the conduct of pandemic research, and implications for ongoing and future critical care research.

Our specific objectives were to understand (1) how national and subsequent local institutional COVID-19 research prioritisation policies influenced critical care research funded prior to the pandemic as well as the conduct of pandemic research; (2) what factors influenced local institutional decision-making around research study continuation, completion and delivery; and what are the implications and lessons that could be learned for future research.

## Methods

### Study design

We conducted a qualitative interview study using semi-structured in-depth interviews underpinned by a Framework Analysis^13,14^ approach.

### Study sample and recruitment

We identified eligible participants from a publicly available list of chief investigators (or their designates) of studies registered on the UK NIHR Clinical Research Networks (CRN) portfolio for critical care studies. Those approached included local principal investigators, senior trial coordinators and research delivery nurses. The UK has a unique infrastructure with the NIHR serving as the research arm to the National Health Service. The NIHR CRN funds and supports regional and local delivery of clinical research. The NIHR has 31 national specialty groups with a remit to focus on coordinated delivery of trials in that specialty. Invitations to participate were circulated via the publicly available contact details of study chief investigators, and via the UK NIHR Clinical Research Network National Specialty Group for Critical Care.

### Sample size

We aimed to recruit a purposive sample of approximately 20 participants, reflecting chief investigators/designates and research delivery staff from various UK centres and representing studies using both interventional and observational designs. We considered this participant number sufficient to gather a broad perspective and to provide enough ‘information power’ given we had a focused aim, a predefined sample, and were drawing on existing theory from similar work on critical care research.^15,16^ The concept of information power considers that the more information the sample holds relevant for the actual study, the lower the number of participants needed.^17^

### Ethical approvals

Ethical approval was attained from the Research Ethics Committee of King’s College London (Minimal Risk Registration Number: MRA-21/22-28426). Participants were provided with an information sheet ahead of the interview, informed digitally recorded consent was taken prior to interview commencement.

### Data collection

We conducted telephone interviews using a semi-structured interview guide (See Supplemental File 1). Interviews were digitally recorded, professionally transcribed and de-identified to ensure anonymity. We sought data on timelines for UPH priority studies from the NIHR and from study investigators to further contextualise our findings. Interviews were conducted by two team members GOG and NP who are clinical academic researchers and critical care nurses.

### Data analysis

We adopted a Framework Analysis approach,^13,14^ which is suited to research with specific questions exploring a pre-determined sampling frame, in this case critical care researchers who were active in COVID19 research, and building on existing work (using a similar sampling approach, as described above) on barriers and facilitators to critical care research.^15,16^ We followed a five-step process: (1) familiarisation; (2) identifying a thematic framework; (3) indexing; (4) charting and (5) mapping and interpretation.^14^ Following data familiarisation, key ideas and recurrent themes were noted with a thematic framework identified from emerging themes or issues. This preliminary framework was then used to filter, sort and classify new data. Subsequent indexing and charting involved labelling, identifying and sorting data according to emerging themes, developing and refining the thematic framework, finally mapping phenomena, finding associations and providing explanations. Following continual review and refinement in the study team, sub-themes and main core themes were revised. Data analysis was conducted by GOG and NP with thematic development and refinement discussed within the broader research team.

## Findings

We interviewed 22 participants from February 2022 to August 2022; (see Table 1 for participant information).

**Table 1.** Participant information.

Region	Role				
	Consultant ITU	Consultant anaesthetics	Research nurse ITU	Research allied health professional	Clinical trials manager
Yorkshire UK		1	1		
North-East UK	1	1			
North-West UK	3				
East Midlands UK	1				
West Midlands UK	1				
East UK			1		
South-East UK	1		1		
South-West UK	1				
London	2			1	1
Scotland UK	1	1			
Wales UK			1		
Not disclosed		1	1		

### Key themes

Our Framework Analysis involved seven initial themes: *Organisation factors; Unit factors; Study factors; Resources; Clinician/Individual factors; Patient/Family factors; Contextual factors*. These were refined into six final core themes, presented below. Sub-themes for the core themes with exemplar quotes are outlined in Table 2 (Additional quotes to demonstrate our themes can be found in Supplemental File 1, Table 3).

**Table 2.** Themes and sub-themes.

Theme	Sub-theme
1. Unit, organisation and national factors	• Global increased profile of research • Wavering engagement overtime
2. Study specific factors	• Complexity of studies • Impact on small-scale research
3. Resources	• Difficulties of staffing • Dual roles • Valuing the workforce
4. Individual and clinician factors	• Managing equipoise • The collective response
5. Family and patient factors	• Increased receptiveness of patients and families to research • Staff fear of added family burden
6. Contextual factors	• Impact of media • Research infrastructure • Increased teamwork and collaboration

#### Theme 1. unit, organisational and national factors

This theme encompassed the subthemes of increasing the profile of research, and wavering engagement. Most participants identified that the COVID-19 pandemic raised the profile of research at both a local and national level, and highlighted hospital and staff buy-in for the government’s research prioritisation strategy. The organisational response varied, but through the national research delivery network (the NIHR Clinical Research Network then) there was a greater degree of national infrastructure to support local organisations, even those who were usually less active. From an organisational perspective, this led to engagement from staff and units who were not usually research active:

UK wide, in terms of COVID studies, the impact has actually been massively positive. I think it enabled a lot of intensive care units that had previously struggled to embed research into their daily practice. I think it really improved the profile of research in critical care in general (Participant ID 10)

However, there was still variation, as while there were national priorities, some organisations prioritised critical care research differently, such as promoting and prioritising local investigator-led research. The pace at which study results were seen, driven by fast-tracked approvals and study implementation, subsequently bought about a rapid change in practice (e.g. dexamethasone use following the RECOVERY trial^4^). Participants perceived this to beneficially affect the wider perceptions of critical care research among their colleagues not usually involved in research activity.

I’ve always struggled to get buy in from my clinical colleagues around trials. But I think that. . .the rapid kind of results [from COVID trials], and then implementation into clinical protocols, clinical guidelines of treatments that came from trials. . . You know, it was very, there’s a really nice story [in that], isn’t there (Participant ID 2)

The situation did present an opportunity for people less familiar with research to understand what it was like to participate, and to be part of large-scale research carried out efficiently and in a coordinated manner.

It demonstrated to everybody the importance of research, you know, to our public and our patients exactly how important research is because I think research was the only thing that offered hope. So, I think that’s a massive gain. I think a huge proportion of patients took part in that research, and therefore their experiences of research have changed and hopefully there’s, you know, we’ve got a bigger patient base for research going forward (Participant ID 4)

Most participants identified that the intense media coverage of critical care resulted in greater public awareness of research, at a level previously unseen leading to increased interest in research participation.

There was a lot of media focus on what was going on in intensive care units. So, people had a sense of what happened there. . . science became much more part of the public discourse, whereas a long time ago we really struggled to message that (Participant ID 9)

Conversely, some participants expressed that some members of the public developed a deep mistrust of research as the pandemic progressed in response to government messaging, media reporting and social media. Fewer families and patients consented to COVID-19 research as time went on.

[Engagement] has been replaced as we go along in a certain cohort with cynicism about the vaccine and cynicism about the therapies, and even relatives pushing us to use, ivermectin for example, in COVID. That’s a small proportion . . . who have swallowed some of the misinformation, who are now actively against research (Participant ID11)

#### Theme 2 study specific factors

Participants outlined the complexity of studies, how the drive for more platform trials could negatively affect sustainability of smaller-scale research, and how large-scale trials could prove challenging for research staff to support if not properly resourced. Participants referred to challenges of managing platform trials, associated trial education, complex interventions and repeated protocol amendments, which some participants described as unsustainable. Furthermore, delivery of complex research interventions competed with the need to train staff on new COVID treatments, creating tension:

The burden of the intervention - it required education of staff to know how to deliver it correctly . . . our education team were swamped with teaching people how to manage COVID, they couldn’t then also teach them how to manage new [research] interventions. (Participant ID15)

Despite these challenges, most participants viewed platform trials as a preferred future model for critical care research whilst acknowledging the associated complexities and expense of platform studies. Reported reasons for this included: perceived efficiencies in trial design, collaborative and consortia approaches to research, and achieving faster results with fewer participants. Conversely, some participants expressed concern regarding the impact of such trials on early career researchers, innovation and less complex research:

What my worry is that it will stifle innovation, recycle [divert] new researchers to things like [platform studies]. If that stays as it is, is that going to be the only study in critical care on community acquired pneumonia. And if so, how do we allow our new researchers to feed into that and get some experience of being a researcher or being a CI [Chief Investigator]. (Participant ID19)

#### Theme 3 resources

This prominent theme encompassed subthemes of staffing (research and critical care), roles, and valuing the research workforce.

##### Staffing

Most participants described staffing for research and clinical purposes as challenging, dynamic and constantly evolving. It involved reassessing risks around reduced staffing (redeployment or recruiting in COVID-19 critical care areas), managing significant staff absences, and balancing clinical and research needs. Whilst many research staff were redeployed initially for clinical work, once the government’s research prioritisation agenda was established, research staff were reallocated to facilitate research delivery. Some participants identified tension arising from conflicting agendas of hospital management and government priorities around maintaining research delivery, particularly for those research staff that were funded through the NIHR CRN.

Staff buy-in for UPH research was viewed as high, given the rapid translation of results into clinical practice identified in Theme 1, and was underpinned by staff factors including teamwork, empathy for the experience of colleagues, with immense goodwill and personal commitment:

The research nurses have been incredibly enthusiastic and that kind of made people bounce off each other and that helps spur it on. (Participant ID1)

##### Clinical and communication role of research nurses in critical care

Witnessing and supporting the unique experience of patients and families at this time, brought with it emotional challenges. Some research staff were also involved in the clinical care of patients.

When I think of [REMAP-CAP], it has personal meaning to me because not only did I recruit, but I nursed those patients as well and I still remember, when I see their names on my patient files, I see their faces and I hear what they said to me ‘am I going to die? Please don’t let me die’, that kind of stuff. So, it’s quite emotive on me as well. (Participant ID20)

Research communication with family members for study information sharing and consent conversations switched to virtual methods, due to local and governmental visiting restrictions, and was considered successful and a positive legacy.

I think in terms of streamlining processes in being able to have consent forms online, take telephone consent . . . email information sheets rather than posting out. Those sorts of things that previously you kind of struggled with; that has helped a lot. And I think . . . I hope to see electronic consent becoming a much more common thing. And I think COVID will have helped that. (Participant ID10)

Overlapping with Theme 5, virtual contact with families was a facilitative factor. Virtual contact and phone management of some processes (such as telephone consent) with families was welcomed and adapted to with ease, although a need for tailored approaches, appropriate to the complexity of the study (e.g. GenoMICC had a simple telephone script as a simple blood test study), was acknowledged.

##### Valuing the research workforce

All participants described a closer collegial relationship between research and ICU clinical teams with research viewed as a credible and positively challenging career option to critical care staff.

. . . we have got more nurses and AHPs that are interested in doing research. I think most people that I have interviewed for posts across the directorate since COVID have a piqued interest in research because they saw what happened during COVID with research, or they were deployed for a while within an area where they say research happening. (Participant ID12)

However, consequences of doing months of overtime, including staff burnout, were described by some of the participants. This included the difficulties of carrying out usual clinical activities in the pandemic, alongside the cognitive burden of learning procedures for several new research studies, and applying new research into rapidly-changing practice at pace. Furthermore, the dynamic impact on skill mix and education was outlined. These factors led to career and life re-evaluation with greater focus on personal needs as opposed to facilitating research. They also led to high levels of staff attrition in critical care.

There is still a lack of some doctors wanting to participate. And I think because they’re so tired after the pandemic . . . And so, the amount of doctors who were on our delegation log almost halved. (Participant ID22)

#### Theme 4 individual/clinician factors

Overlapping with study factors, this theme arises from issues around how specific studies were chosen for participation and received at sites. Some participants reported clinical equipoise as being questioned increasingly, particularly for ongoing platform studies.

And you know, we’ve got mixed views then because some of my colleagues say we just shouldn’t randomise to interventions we think might be harmful. So, we didn’t open [a particular arm] in that study for that reason and subsequently were proven to be correct about that. (Participant ID16)

Individual factors also encompassed pride, as a result of personal, local and national involvement in research during the pandemic.

It gave them [clinicians] a bit of hope, a bit of sort of, you know, a positive feeling, they were contributing something other than just the care of this particular patient. So there was a real psychological benefit to the clinical teams being involved [in research] . . .they felt that really strongly . . . because of the psychology involved, we felt that was a greater good commitment. (Participant ID16)

Some participants described how some junior staff had formative career experiences of pandemic research. The collective response made the scale of the research delivered in the midst of the pandemic possible.

We got thousands of patients through with a sort of whole Trust [hospital] response, you know, there were hundreds of doctors volunteering, there were all kinds of PhD students, and this sort of ramp up of that took about two weeks . . . that was key. (Participant ID1)

#### Theme 5 family/patient factors

Despite changes in some public perceptions over time, noted in Theme 1, most participants highlighted the positive response of patients and family members to research. There was an increase in engagement, knowledge and receptiveness to taking part in research, contrasting with pre-pandemic experiences, further highlighting the notion of contributing to the ‘greater good’.

. . .the patients and their relatives were just receptive. We want to find anything that can help this pandemic. And so, unlike any other studies ever done, at the beginning the amount of people refusing to be in studies was zero. (Participant ID22)

However, the inability to visit (particularly in wave 1) was an important factor for families, and the drive to recruit as many participants as possible left some staff feeling uncomfortable having to contact families about research studies:

. . .they couldn’t come in to visit their relatives, which was horrendous for them anyway, everything was done over the phone or via FaceTime. It was really impersonal and very anonymous . . . And we all felt really uncomfortable because you kind of felt like you were cold-calling people. . . (Participant ID 10)

Research staff also had to reassure clinical staff that taking part in research would not add further burden for families who were already under significant stress.

. . .one of the things that the non-ICU research nurses struggled with was that they worry about the burden of the trial. But actually, patients and their families cope very well with being approached about studies in a very difficult time. . . (Participant ID 2)

#### Theme 6 contextual factors

The impact of media, R&D infrastructure and clinical capacity on the research response, and the associated increased teamwork and collaboration provided a backdrop against which critical care research was being conducted. This interlinks with Theme 1, which encompassed the national factors affecting how trials were delivered and prioritised. For instance, media profiles for critical care research were high, as a result of national interest, which meant resources being temporarily diverted to critical care research, and governance processes being simpler. However, while these measures were broadly welcomed, participants acknowledged such processes as being time and context-dependent, and that ongoing maintenance of these efficiencies would be unsustainable. While there was a temporary increase in R&D infrastructure and support, the unprecedented backlog in clinical treatments (such as elective surgery) grew, and these were viewed as a both positive and negative legacies of COVID-19 on critical care research. There was greater opportunity and resource to focus on surgical studies in the aftermath of the pandemic.

The volume of patients coming through for elective surgery has greatly stepped up, which means that it’s a good time now to do the studies that are focusing on improving perioperative outcomes. . . . So . . . hopefully we can maybe . . . those studies will recruit quicker hopefully, and answer key questions quicker (Participant ID4)

Transition and recovery of usual clinical activity swiftly became the new focus. Some participants outlined how clinical staff were motivated to continue to engage in research activities, and to continue to deliver research at pace, using the relationships between research and clinical staff developed during the pandemic.

I’d like to think that we can still maintain the relationships that were built up during COVID, of collaboration, both internally and externally. I hope we can continue to deliver at the level we have been able to deliver at. . . (Participant ID12)

Several expressed concerns about diminished funding and lack of a national or local strategies about how to prioritise which critical care research to resume.

there was then no strategy either from the Trust [hospital] or nationally about what would restart. The NIHR did come up with criterion, but they were relatively loose, and it was really down to individual Trusts about how they would classify whether it was a priority and middle priority or a low priority. (Participant ID8)

## Discussion

Our study identified several key issues. These relate to individual teams within organisations, and the type of research, in terms of how research was viewed during the pandemic, including the legacy this has had on how clinicians engage with critical care research going forward. Additionally, sharing early information about the progress and clinical impact of trials was seen as crucial, such as the cessation of treatment arms when deemed ineffective. It also extended to workforce and engaging clinical staff in research activities, and rotation of research staff into clinical teams. The UK benefits from a unique infrastructure, the NIHR, to support trial delivery of funded research. It is clear from our work that harnessing the momentum achieved in the collective research response in critical care during the COVID-19 pandemic is key to sustain clinician engagement and better collaborative working between research and clinical teams, so that research is normalised as part of the continuum of clinical care.^15,16^

Long-term impacts of the research and clinical activity during the pandemic have begun to emerge, including the feasibility of sustaining research activity for national studies that were unfunded, or insufficiently funded, during the pandemic, and addressing ongoing clinical backlogs and the implications of prioritising studies that can answers questions rapidly at scale, such as platform studies. Although several studies were forced to closed down,^18^ impacting careers and future research programmes, our data suggest there were enhanced research opportunities that many researchers, trainees and clinical staff capitalised on during the COVID-19 pandemic to develop their research capacity and skills.^18,19^ Our findings should also prompt the critical care research community to consider how to best meet the needs of the future critical care workforce, who may need to respond to future crises through research. Possible solutions for this include, preparing ‘sleeper’ study protocols that can be enacted swiftly during pandemics,^20^ alongside developing training frameworks for rapid deployment of staff into research and creating local and national procedures for fair and transparent prioritisation during crises situations. Moreover, a positive legacy noted in this study was the viewing of research as part of the clinical continuum during the pandemic, something the authors have previously argued for.^21^

The collective, national response, certainly provided hospitals and research teams with a mandate to prioritise research in critical care. Our data also points to the legacy of prioritising studies, meaning important studies were stopped, never to restart, with concomitant resource implications. There were also accusations of research waste during this time, particularly in the number of COVID-19 trials that were not completed, or that duplicated existing studies,^22,23^ and ultimately this means individual patients’ participation was wasted in many cases. The UK’s collective and coordinated approach was heralded during COVID-19, and continuing this tactic, in the context of more scarce R&D and staffing resources, requires thoughtful engagement with the critical care research community to identify how to maintain a collective effort. We identified that research active clinicians had to manage a substantial and extended increase in both their clinical and research workload, creating a legacy of burnout in some should not be underestimated or forgotten.^24,25^ Formalised structures around working patterns and protecting staff need to be considered for future pandemic and staffing crises.

The future of critical care research is informed by the COVID-19 response to research; our data point to the advantages of gaining knowledge to inform treatments, swiftly and through coordinated, efficient research studies. For example, capitalising on the success of platform trials during the pandemic is important, while also acknowledging some of the unintended consequences of platform trials,^26,27^ including the risks to smaller studies needed for innovation, or studies of healthcare systems as well as implications on research opportunities and careers. Additionally, our data described the lessons learned about benefits and risks of study prioritisation as well as how digital or virtual mechanism can support research delivery and collaboration. We should take advantage of the momentum gained including different research models adopted, for instance in terms of informed consent procedures, which should be continued outside of pandemic conditions to optimise research for patient benefit.^28^

Limitations of the study include the qualitative nature of the study with all participants directly engaged (and therefore invested) in critical care research. A different perspective may have emerged by recruiting participants not engaged in research. Although we sampled across the UK, we may have missed areas of low research engagement and uptake. The family and patient perspective is missing. They may have had important insights for implications for critical care research contingency planning for future pandemics.

## Conclusion

Our study emphasised the value of a collective, coordinated response to pandemic research in critical care and how this helped draw together the research workforce to deliver timely trials to answer complex clinical questions about the COVID-19 treatments. However, there were implications of national prioritisation, including cessation of important non-COVID-19 studies. Future research in both pandemic and non-pandemic situations, should focus on formalised preparations to manage not only studies and prioritisation, but also the workforce response, and engagement of clinical teams. As and when scaling up is needed, having large-scale infrastructure ready to be operationalised is crucial to contingency management and the delivery of research for responding to pandemic need.

## Supplemental Material

sj-docx-1-inc-10.1177_17511437241301921 – Supplemental material for The legacy of the COVID-19 pandemic on critical care research: A descriptive interview study Supplemental material, sj-docx-1-inc-10.1177_17511437241301921 for The legacy of the COVID-19 pandemic on critical care research: A descriptive interview study by Natalie A. Pattison, Geraldine O’Gara, Brian H Cuthbertson and Louise Rose in Journal of the Intensive Care Society
