# Supplementary material for: The legacy of the COVID-19 pandemic on critical care research: A descriptive interview study
Source: J Intensive Care Soc. 2024 Dec 8;26(1):53–60. doi: 10.1177/17511437241301921 (PMC11626551; doi:10.1177/17511437241301921)
Supplement: sj-docx-1-inc-10.1177_17511437241301921 – Supplemental material for The legacy of the COVID-19 pandemic on critical care research: A descriptive interview study [file sj-docx-1-inc-10.1177_17511437241301921.docx]

| Theme | Subtheme | Exemplar quote |
| --- | --- | --- |
| 1.Unit, organisation, and national factors | - Global increased profile of research - Wavering engagement over time | *‘…because the studies are being publicised on BBC news in the evenings . . . people actually understood it and people understood the importance of the research. And that was true of the clinical staff as well’ (11*)  *‘There has been a contraction in the amount of funds that have been made available to research across the board since COVID. But, I mean, I think, I don't think that was sustainable. I don't think that the money that COVID brought to medical research could have been sustained’ (14)* |
| 2.Study specific factors | - Complexity of studies - Impact on small scale research | *‘X study particularly has become extremely complicated with all the different...I mean it's become a full- time job for our research nurses just to get their head around the various contraindications and interactions between the domains ...it’s like 16 clinical trials within one’ (11)*  *‘The first trial we started . . . was really, really complicated. Units that are naive to research would struggle with some of the more complex trials’ (11)*  *‘I think there is a danger that the UK community would just say, okay, well, we're now only gonna do one sepsis study, and so everybody else's sepsis study can go by the wayside’ (8)*  *‘The smaller ones really had to stop. Especially for the people doing their own PhD studies, or smaller studies, and they were the sole person recruiting and analysing data’ (6)* |
| 3.Resources | - Difficulties of staffing - Dual roles - Valuing the workforce | *‘...most of our research nurses are actually highly qualified individuals that can provide care. So, they are a major resource in being able to do clinical shifts . . . if you had critical care skills and can look after a ventilator and you are a research nurse, you are hot property to be working in critical service…’ (3)*  *‘… to expect a team to run a multi-platform drug study in the middle of trying to actually give care to more patients than you would normally, I think you've got to then give more research staff to that area’ (20)* |
| 4.Individual and clinician factors | - Managing equipoise - The collective response | *‘We stopped recruiting to, because of the lack of equipoise for the remaining arms, I suggested that we would pause it, that we'll stop it and sort of have a rethink. So, we did that, and we didn't continue with it’ (20)*  *‘Suddenly it was like, oh no, actually that's fine, we'll get it done. And we've had buy-in, for example, from the pharmacists as well (1)* |
| 5.Family and patient factors | - Increased receptiveness of patients and families to research - Staff perception of added family burden | *‘I think it's been easier with the pandemic, to get more engagement from patients . . . And in fact, on reflection, we did actually have patients asking, specifically for COVID, they were aware of what trials were being, that were running, and treatments, and they did actually on occasion come forward and mention these trials, which was very different from obviously the past’ (5)*  *‘…particularly in the first wave, you know, their families would be so destroyed that you wouldn't ring them at home to say just, you know, we put them in this research trial, because you would be adding to their distress…’ (10)*  *‘I think the fact that the relatives couldn't be at the bedside or face to face, probably created a whole area of stress and uncertainty for relatives which is, I'm not sure if we understand that yet’ (13)* |
| 6.Contexual factors | - Impact of media - Research infrastructure - Increased teamwork and collaboration | *‘I think people do value research, but I think you know, that COVID has kind of put it on a plate and put it in front of them. And, you know, you can't ignore the impact that research has had on saving lives’ (4)*  *‘I think it was a very streamlined, you know, the, usually sort of, trials take a long time, relatively a long time to go through the approvals, etc, etc, and X study demonstrated that, you know, a streamlined way of getting approvals is you know, by using that approach, you can still deliver safe research quickly’ (4)*  *‘It’s the goodwill of the R and D department. It's the goodwill of our administrators. It's the goodwill of our research nurses and it's the goodwill of our PIs who, you know, come in at weekends when they shouldn't be to recruit patients. Who, you know, stay late at work, who go the extra mile’ (8)* |
